# Special features of health services and register based trials – experiences from a randomized trial of childbirth classes

**DOI:** 10.1186/1472-6963-8-126

**Published:** 2008-06-11

**Authors:** Elina Hemminki, Kaija Heikkilä, Tiina Sevón, Päivikki Koponen

**Affiliations:** 1 National Research and Development Centre for Welfare and Health (STAKES), Helsinki, Finland; 2 Laurea Polytechnic, Espoo, Finland; 3 National Public Health Institute, Helsinki, Finland

## Abstract

**Background:**

Evaluating complex interventions in health services faces various difficulties, such as making practice changes and costs. Ways to increase research capacity and decrease costs include making research an integral part of health services and using routine data to judge outcomes. The purpose of this article is to report the feasibility of a pilot trial relying solely on routinely collected register data and being based on ordinary health services.

**Methods:**

The example intervention was education to public health nurses (PHN) (childbirth classes) to reduce caesarean section rates via pre-delivery considerations of pregnant women. 20 maternity health centers (MHC) were paired and of each 10 pairs, one MHC was randomly allocated to an intervention group and the other to a control; 8 pairs with successful intervention were used in the analyses (1601 mothers). The women visiting to the study maternity centers were identified from the Customer Register of Helsinki City. A list of the study women was made using the mother's personal identification number, visit date, the maternity center code, birth date and gestation length. The mode of delivery and health outcomes were retrieved from the Finnish Medical Birth Register (MBR). Process data of the intervention are based on observations, written feedback and questionnaires from PHNs, and project correspondence.

**Results:**

It took almost two years to establish how to obtain permissions and to actually obtain it for the trial. Obtaining permissions for the customer and outcome data and register linkages was unproblematic and the cluster randomization provided comparable groups. The intervention did not succeed well. Had the main aim of the trial been to cause a change in PHNs behavior, we would have very likely intensified the intervention during the trial.

**Conclusion:**

Our experiences encourage the use of trials that obtain their outcomes from registers. Changing the behavior of ordinary health service providers is a challenging intervention.

**Trial registration number:**

not registered (see Results)

## Background

Trials are common in evaluating simple health care interventions that have commercial interests, such as drug therapy, but not in the case of trials involving more complex interventions or technologies with no commercial benefits. Obstacles to complex trials include a difficulty to make experimental practice changes, ideological resistance to or ignorance of trials, unknown levels of generalizability, and costs. Ways to increase research capacity and decrease costs include making research an integral part of health services, rather than a separate task to be paid for by researchers, and using routine data to judge outcomes. Collecting outcome data from routine administrative registers may notably reduce costs [1] and allow a complete follow-up. However, the current norms of research ethics approvals [see e.g. [2]] do not encourage such designs.

Registers with personal identification numbers have been widely used in non-experimental health and health service research [1,3,4], and register linkages have been frequently used as a tool for follow-up, and to identify deaths [5]. However, in trials registers have much less been used to measure main outcomes. Williams et al. [1] have made an analysis of the potential usefulness of registers in the United Kingdom as a source of trial outcomes. They hypothetically replicated four earlier trials (that had used specially collected data), and concluded that routinely captured clinical data have real potential in measuring patient outcomes, especially if the detail and precision of the data could be improved. In a preventive drug trial in Scotland [5], register linkages provided a valid method to record adverse events.

Like in other Nordic countries, national health registers using personal identification numbers and having complete coverage and good quality are numerous in Finland [4]; additionally there are many local or disease specific registers [6]. Trials on cancer screening based on ordinary health services have been made that use either individual or cluster randomisation or matching and have obtained all their main outcomes from registers [7-12]. In other individually randomised Finnish studies, registers have formed an additional data source, especially in long-term follow-up [see e.g. [13,14]].

The purpose of this study was to pilot the feasibility of a trial relying solely on routinely collected register data and being based on ordinary health services in the field of maternity care. The example intervention was the further training of public health nurses on prenatal childbirth education to pregnant women (hereafter called childbirth classes) to reduce caesarean section rates via pre-delivery considerations by pregnant women.

The purpose of this paper is to report the experiences of doing such a health services and register based trial. Furthermore, we briefly describe the impact of the intervention on cesarean section rates.

## Methods

The trial context is given in the Appendix.

### Allocation

The trial used matched-pair design. At the time of the study (2002–2003) there were seven health areas in Helsinki city, divided into 32 health stations, each including at least one maternity health centre (MHC). All 20 MHCs in four health areas (three areas did not participate due to other development projects) were paired using the delivery hospital (HUH or CH) and the way of organizing childbirth classes (whether given by the woman's own prenatal PHN or by a PHN specialized in childbirth classes) as primary matching criteria. Additional matching criteria included the size of the MHC, number of women per PHN, and the proportion of pregnant women aged over 34 in 2001. The characteristics of MHCs were obtained from an e-mail survey to the nurse managers of the health stations. One of each of the 10 pairs was randomly allocated to an intervention group and the other to a control group on the throw of a dice. There were 67 PHNs in the intervention and 70 PHNs in the control MHCs.

In addition to our survey to nurse mangers and the PHN survey (see below) in 2002, statistical data of the intervention and control MCHs were available from an unpublished quality measurement report of Helsinki health center in 1999. Based on these data sources, the intervention and control MCHs were similar in regard to type of personnel (in 2002, 15% and 16% had midwife education, mean of experience in maternity centers was 8.3 and 7.5 years) and the main delivery hospital. The control MCHs were, as a mean somewhat larger, measured by the mean number of PHNs per MCH (6.1 and 8.2 in 2002) and the mean number of births per MCH (163 and 200 in 2002). However, the number of mothers per PHN was higher in the intervention PHNs (mean births 34 and 26 in 1999).

### Intervention

The intervention was the further training of PHNs to pay more attention to the mode of delivery in childbirth classes and informational material given to the pregnant women. The intervention consisted of: a) a joint educational session (1.5–2 hours) to all PHNs in the MHC, given by an experienced midwifery teacher (KH) using instructional conversation in small groups; b) a leaflet on childbirth and preparation for it, which PHNs were asked to give to the pregnant women and to discuss its contents both during childbirth classes and other visits from week 32 onwards, c) a file of evidence-based research material on the same topics for each MHC, d) a questionnaire to PHNs on their opinions and knowledge of childbirth to be filled before the educational sessions. All these four items dealt with the benefits of vaginal delivery, the reasons for and consequences of caesarean section, and the possibilities of pregnant women to influence the mode of delivery.

Description of the intervention process is based on observation notes, written feedback from PHNs, PHN questionnaires (60% response rate) and project correspondence. The difficulties encountered with organizing educational sessions to PHNs (see Results) were reflected on and the causes discussed and noted during the study. Six months after the intervention a questionnaire with semi-structured questions was given (by KH) to all PHNs in the control and intervention MHCs during their ordinary staff meetings; 54% of the intervention PHNs responded. In addition to this questionnaire, the success of the intervention was judged by observations made during the educational sessions; notes of the sessions and the feedback given were written down immediately after each session.

### Trial process

We aimed to obtain a supportive statement from a research ethics committee acceptable to Helsinki city health services and registry owners. The difficulties encountered with ethics committees (see Results) were noted during the study.

The hours taken to set up the trial and to get the data were estimated (partly from working time records), and changed into euros using each researcher's salaries multiplied by 1.3 (social security costs); institutional costs were not included. Costs not taken into account included the costs of planning the scientific content, of familiarization within the field situation, of carrying out the intervention (creation of material, educating etc), or the time spent by PHNs in the new childbirth classes.

**Figure 1:**
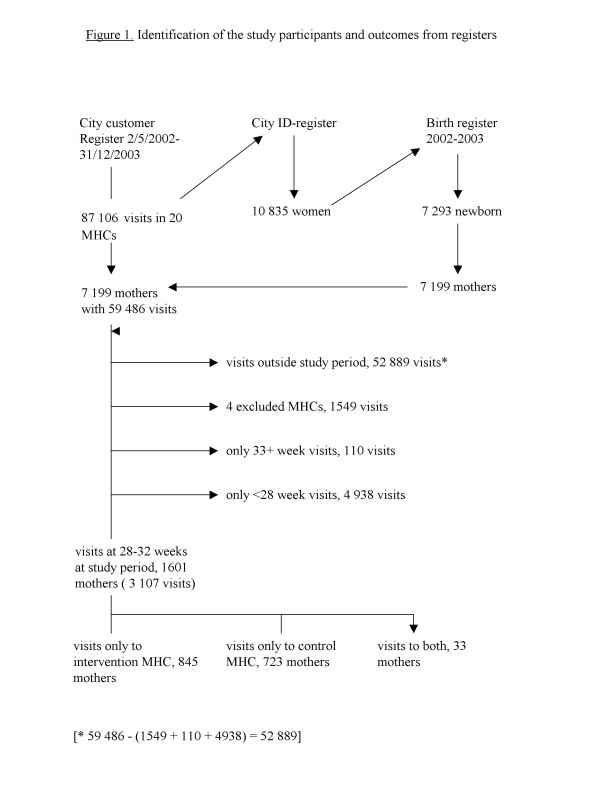
Identification of the study participants and outcomes from registers.

To identify the intervention and control mothers, visits to the study maternity centers in the intervention period from May to December 2002 and also for 6 months after that were obtained from the Helsinki City Customer Register, run by a commercial company (WM-data NOVO), and linked to the Finnish Birth Register (MBR), Figure 1. MBR collects demographic, medical and service utilization data of all mothers and their newborns; after record linkage to the population and cause of death registers, its coverage is complete. The identification code is the mother's personal identification number.

The customer register includes the service unit name, the date, and patient's personal identification number. A list of women having visited the maternity centers during the study in gestation weeks 28–32 period was made using the mother's personal identification number, visit date, the maternity center code, birth date (MBR), and gestation length (MBR). Childbirth classes are usually given around 28–32 weeks of gestation.

No sample size calculations were made because this study was a pilot testing the feasibility of the study design and the strength of the intervention. Because the intervention did not succeed well (see Results), no power calculations were made even later. The background characteristics of the mothers and outcomes in the intervention and control groups were first compared by cross-tabulation, and the statistical significance of the differences were tested by Chi-square and t-tests.

### Outcome data

The mode of delivery and other outcomes were retrieved from MBR. The odd-ratios of the delivery interventions and infant health outcomes were calculated by logistic regression, adjusting for the differing background characteristic (marital status, see Results). Our plan was to compare the groups, besides by simple logistic regression, by multilevel logistic regression to accommodate the cluster effect. However, because the intervention did not succeed (see Results), only basic outcome data were analyzed and cluster effect was not taken into account.

## Results

### 1. Permissions for the trial

It took almost two years from the beginning of the negotiations to establish how to get permissions for the trial and then getting them. Finland had new legislation relating to ethics committees in 1999 requiring "medical" research to be approved by a "certified" ethics committee. However, for interventions like ours it was not clear whether it was medical research as legally defined, other health research or developing health care, and what kinds of permission were required. After several discussions and meetings between the body coordinating research activities within Helsinki City health services (TUTKA), city health planners, and the people responsible for developing prenatal care in April–November 2000, we submitted our study to the Hospital District of Helsinki coordinating ethics committee in December 2000; Helsinki City no longer had an ethics committee of its own. Because the primary health care ethics committee had not been established, the coordinating committee transferred our application to the gynecology and obstetrics committee within the Helsinki University. That committee twice asked for clarifications, mainly on the education content and the people involved in the research, but did not give a decision.

After eight months, TUTKA, on our plea, decided that the project (not including data collection) is development work and no ethics committee statement is required. After four months (in January 2002) permission to carry out the intervention in four out of seven health areas was given by the City Health Director.

The research part of the project was handled and approved by STAKES research ethics committee (October 4, 2004). Applying for and obtaining permission for the customer and outcome data (MBR), and obtaining an ethics committee statement went without problems.

### 2. Success of intervention

The City health administration (chief nurse manager) helped to contact the nurse managers responsible for PHN work in the MHCs, and a presentation in their monthly meeting was given. The responses varied among the nurse managers to our requests to meet the PHNs, to give an educational session and to distribute the material. Even though all agreed that the development of childbirth classes is important, many complained of too many development projects and too many studies being carried out in MHCs.

Two MHCs did not want to take part in the intervention, mainly due to lack of time. They and their pairs were excluded from the analyses, leaving 16 health centers (8 pairs). The responses of PHNs in the educational sessions varied. Only 40 of the 67 intervention PHNs attended the education; other PHNs had conflicting appointments or were off work; some temporary staff did not attend either. Based on observations as well as on the answers given in the PHN questionnaires, our interpretation is that most PHNs were in principle interested in childbirth classes, but the classes were not the priority among the many tasks they had been given and needing their attention. They thought that caesarean sections and other surgical deliveries were hospital obstetricians' decisions and that pregnant women were advised and consulted by delivery hospital staff.

### 3. Identification of the study groups and outcome data collection

The city health services and data keepers were sympathetic to our requests to identify the study women. Carrying out the register linkages were easy in principle (uniform personal identification number), but the change in the data system in the Customer Register during the study period resulted in technical complexity and extra work. We calculated that following hours were used: negotiations with the city health administration 66, application for and negotiations with the Customer Register 18, applications for and negotiations with the hospital ethics committee 27 and STAKES ethics committee 21, data extraction from the Medical Birth register 4, data linkages and quality checks 42, total 178 hours. We do not know the number of hours used to extract the data from the Customer Register, but the bill was 1862 euros,

In total 1601 mothers had visited the 16 study MHCs (2 pairs were excluded, see Results) at 28–32 gestation weeks during the study period (Figure 1). Most (96.8%) had visited only one maternity center, or had visited only intervention or only control centers (1.1%); 33 (2.1%) mothers had visited both an intervention and a control center, and were thus excluded.

### 4. Comparability of the groups

Measured by mothers' background characteristics, the cluster randomization succeeded relatively well (Table 1). There were more women in the intervention than control groups, but with the exception of marital status, the pregnant women's background characteristics were very similar. In addition to the variables in Table 2, we studied the distribution of the number of previous pregnancies and the time of the first prenatal visit (no differences).

**Table 1:** Comparability of the intervention and control groups, % if not otherwise indicated^1)^

	**Intervention**	**Control**
	
	(n = 845)	(n = 723)
Age at birth, mean (SD)	30.6 (5.5)	30.6 (5.4)
< 25 yr	14	13
25–29	28	30
30–34	30	31
35 +	27	27
Marital status		
Married	52	59
Common law	38	35
Other + NG	10	6
Social class		
Upper white collar	27	29
Lower white collar	31	29
Worker	10	9
Student	13	12
Other + NG	20	20
Previous births^2)^, mean (SD)	0.67 (0.97)	0.70 (0.98)
0	57	55
Previous pregnancies^2)^, mean (SD)	1.07 (1.27)	1.16 (1.32)
Smoking^2)^		
No	84	85
Current	14	13
Quitted (during pregnancy)	2	2
Timing of 1st prenatal visit, mean (SD)	9.0 (3.1)	9.0 (3.1)
< 10 gest. weeks	69	68
Boy	53	52
Twin	1	1
Pregnancy length, mean weeks (SD)	40.1 (1.6)	40.0 (1.6)
< 37	3	3
37–41.9	89	91
42+	8	6
Birth weight, mean g (SD)	3521 (487)	3522 (537)
< 2500	3	4
2500–2999	9	9
3000–3499	35	33
3500–3999	40	36
4000+	13	18

^1) ^NG = no information

^2) ^Missing information 0–4 women in each group

**Table 2:** Interventions during labour and delivery and infant outcome^1)^.

			**Crude**	**Marital status adjusted**^1)^
	
	**Intervention**	**Control**	OR (95% CI)	OR1 (95% CI)
	
	(n = 845)	(n = 723)		
Mode of delivery	%	%		
C-section (any)	19	16	1.29 (0.99–1.67)	1.29 (0.99–1.68)
Planned	7	6	1.18 (0.78–1.79)	1.20 (0.79–1.82)
Other	13	10	1.31 (0.95–1.79)	1.30 (0.95–1.78)
Instrumental	7	6	1.05 (0.70–1.56)	1.01 (0.68–1.52)
Vaginal	74	78	0.81 (0.64–1.02)	0.81 (0.64–1.03)

1) Crude odds ratios (95% confidence intervals, CI) and odds ratios adjusted for marital status and birthweight. In adjustment for marital status classes used were: married, common law, other.

To estimate caesarean section rates prior to intervention, we used the data of women who were past 32 weeks when the intervention started, and thus unlikely to get the new childbirth education. The caesarean section rate was higher in the intervention maternity centers (15% of 231 women) than in the control maternity centers (12% of 219 women), OR 1.23 (95% CI 0.71–2.11), but the difference was not statistically significant.

Pregnancy care, delivery hospital, and pregnancy length were similar in the two groups. The distribution of the delivery hospitals was the same in the two groups (61% in the City Hospital, 36% in the University Hospital and 3% elsewhere). The mean birth weights were very similar.

### 5. Effectiveness of the intervention

Caesarean sections were somewhat more common in the intervention group, but not statistically significant, Table 2. Had we correlated for cluster effect, the confidence intervals would have been even larger. Neither were there any statistically significant differences in operative deliveries or other delivery procedures or in infant health. We studied pain relief, labor induction, use of oxytocin, fetal electronic surveillance, Apgar scores, care in neonatal or in intensive care units (a subgroup of care in neonatal units), newborn resuscitation, and perinatal and infant deaths (4 deaths in each group).

When only the first-time mothers were included, results similar to those of all women were obtained. The difference in caesarean section rates was not statistically significant (odds ratio adjusted for marital status and birth weight was 1.30, CI 95% 0.93–1.82).

## Discussion

In this trial, the feasibility of obtaining outcome data was shown to be unproblematic and could be easily and economically obtained from existing registers. To identify the trial subjects we used a register which had not previously been used for such a purpose, and some extra work resulted from the novelty. Future trials would very likely be easier. The intervention based on health care services turned out to be weak.

A strength of the study was that it evaluated the use of routine data in a real trial. Furthermore, the process data were prospectively collected as part of the study. Record linkages and other IT work were done by experienced personnel. Thus our study – even though the first of its kind in Finland using Medical Birth Register and Helsinki Customer register – gives a realistic estimate of the work load if health registers were routinely used as a data source.

However, the resource estimates were somewhat arbitrary and we had no comparison, i.e. what the costs would have been if we had identified the women, approached them, and collected the outcome data either directly from them or from patient records. However, our experience from traditional trials suggests that the outcome collection costs in this study were small. We limited the estimation of the used resources narrowly, because we wanted to study whether the costs of data collection and setting up a trial could be made cheaper. These results are more generalisable than intervention costs, which are always bound to the type of intervention. But separating the "trial set up" and "intervention" costs was difficult: a mapping of the situation and negotiations could serve both elements.

The number of hours used for planning data collection, and negotiating and getting permission was reasonable. How applicable are these results to other countries or other types of trials? Because the rules governing research and salaries vary, the costs may not be directly transferable to other countries. However, the magnitude, the costs being notably smaller than if data had been collected in a traditional way, e.g. from patient records, is likely to be true also in other countries having registers suitable for research. In our example, only two relatively simple registers were used. The use of more complex or just more registers would likely lead to higher costs.

The possibility to use registers depends on their existence, content, and availability. In Finland, all citizens have a unique personal identification number (ID-number), there are numerous health registers, and well-developed data protection laws allow research to be made in a controlled and ethical manner [4]. The completeness and data quality of the key health registers, including the Medical Birth Register, are good [15]. The validity of the variables used in this study is good [16]. The quality of customer registers in health care has not been studied, but is assumed to be good due to administrative and financial implications. Countries not having wide use of personal ID numbers or having less reliable health registers or less developed data protections laws may find the use of registers less appealing. However, the wide use of registers in epidemiological research (for perinatal period, see e.g. [17,18]) suggests that registers could be used for trial outcomes also outside the Nordic countries.

However, the kinds of outcomes which are available from the birth register are limited, and no subjective data, such as satisfaction, are collected. This limitation is likely to remain, because adding subjective information into routine registers – even though technically easy – is likely to limit register use in research, as requirements for written informed consent may become a prerequisite.

In principle, registers and other routinely collected data can be used to measure outcomes both in traditional "on invitation" trials and trials based on ordinary health services. In the former, if that possibility is thought through ahead of time and informed consent requested, registers are a useful data source, providing follow-up data of all participants without recall, selection, or participation bias [5]. However, if the follow-up of the trial participants and late outcomes is an after-thought, data protection laws and other research rules may prevent it without first obtaining a new informed consent.

Trials based on routine health services, i.e. where it is prospectively decided to provide services differently to similar patients (or healthy people in the case of preventive trials), are much rarer than trials based on invitation and informed consent. In trials based on routine health services, outcome measurement from routinely collected data may be the best or only feasible method. If such trials become popular, as we think they should, it is likely to put further pressure on the need for good data protection laws and the quality of registers.

The intervention part of our trial was, after long deliberation, defined by the research governance system to be "developing work" rather than "research"; only the data collection part was defined as research. These definitions had a crucial impact on feasibility. The current Finnish research regulation is unclear in terms of health-services-based research, complex interventions, and cluster randomization, and research ethics committees have difficulties in applying the regulation [2]. That our intervention was evaluated to be development work did solve the problem. However, to encourage future health-service-based and cluster randomized trials for complex interventions, research regulations should be modified to accommodate the special needs involved. For example, individual informed consent is not sensible for a randomized invitation to cancer screening or for consulting health care providers who have been randomized to offer varying services.

Our original idea of the intervention was stimulated with a suggestion that information to women might contribute to appropriate CS rates [19] as well as by the general discussion of women's wishes for caesarean sections [20]. We thought that in the Finnish context, the natural persons to do that would be the PHNs in prenatal care, who also otherwise help woman to meet the new requirements of motherhood. Mechanisms through which women can influence the mode of delivery may be their general attitudes and wishes before labor.

The intervention – education of public health nurses (PHN) to empower women to avoid caesarean sections with relative indications – did not succeed very well. We noticed that the intervention was weak already during its implementation. Had the main aim of the trial been to cause a change in PHNs behavior, we would very likely have intensified the intervention, for example, by further educational sessions or discussions with PHNs and their supervising general practitioners, as well as closer collaboration with the delivery hospital personnel. Because the intervention was weak, an impact on outcomes was unlikely.

Why did the intervention remain weak? One reason may be that Helsinki PHNs do not consider delivery as their area of expertise, but that of hospitals. Due to the multitask work [21], some PHNs had very few pregnant women to look after, and even among those having more mothers, childbirth classes were not a priority. Furthermore, in Finland, there has been a clash between midwives and PHNs about their roles and competence within prenatal care [21-23]. Our intervention was brought to bear externally and by a midwife teacher; maybe the PHNs might have needed someone from their own organization or profession. Secondly, maybe practicing PHNs were not accustomed to think that women's empowerment or decision-making would be important in delivery. Further reasons may have been that PHNs were tired of the many development and research activities being carried out in Helsinki prenatal care, with any new program meeting with some resistance

This may describe the likely scenario more generally: bringing systematic and selective changes in the way services are provided so as to enable reliable evaluation is the difficult part, while measuring the impact from routine data is perhaps easier. The content of and the way in which health services are provided is an amalgamation of tradition, research results, interest-group influence, politics and costs. Making changes which researchers would like to see happen requires both health professional and political support. After such support is given, a randomized introduction may not be feasible, because decision-makers are eager to have the new service provision to all units. This may be the case even when a totally new service is launched, and even more so when existing services are modified. Differing and untested technologies and services can be used as long as their use is individually decided by professionals or patients. But the systematic offering of different services to allow proper comparison is not yet customary and hindered by current practices of research and service regulation [2].

## Conclusion

Our experiences are encouraging for the use of trials designed to obtain their data from registers, though research ethical rules need to be clarified. If the intervention is based on changing the behavior of either the service provider or patients/people, the difficulties in implementing these changes, and then only in the intervention group, are likely to remain a challenge.

## Appendix

Trial context and the importance of the intervention.

The trial was carried out in maternity health centers (MHC) in Helsinki, Finland. These health centers provide prenatal care as part of primary health care. Services are organized by areas, but if a woman moves during pregnancy, she can visit the maternity center of her old area. Delivery care is a part of specialized obstetric hospital care. Most births occur in the two maternity hospitals (Helsinki University Hospital [HUH] and City Hospital [CH]). The delivery hospital is determined by the woman's place of residence, but complicated cases are referred to HUH. Women can also apply for hospitals outside Helsinki.

Prenatal care is run by public health nurses (PHN), formally supervised by general practitioners. Most prenatal care providers were solely PHNs, but some had also specialized in midwifery. Both PHNs and midwives (subsequently called PHNs) are well educated, with 3.5–5 years of education after high school. Traditionally, preparation for birth has been an important task of PHNs. The organizational separation of delivery and prenatal care and the scant education of younger PHNs in delivery care have, however, resulted in ambiguity. In Helsinki, the volume and content of childbirth classes varies by MHCs, and an internal document by City of Helsinki Health Centre in 2001 on prenatal care called for high-standard, uniform childbirth classes. Caesarean section rates in Helsinki have been much higher than in Finland on average, with internal hospital scrutiny being focused on the matter in recent years. Thus, there was an interest within the city's health administration in the substance of our trial.

## Declaration of competing interests

The authors declare that they have no competing interests.

## Authors' contributions

EH designed the study and wrote the first draft. KH did the intervention and collected the qualitative data of the trial process. TS did the register linkages and analyses. PK supervised the intervention and participated in writing the qualitative part of the manuscript. All authors read, commented and approved the final manuscript.

## Pre-publication history

The pre-publication history for this paper can be accessed here:


